# Cell membrane fatty acid changes and desaturase expression of *Saccharomyces bayanus* exposed to high pressure homogenization in relation to the supplementation of exogenous unsaturated fatty acids

**DOI:** 10.3389/fmicb.2015.01105

**Published:** 2015-10-12

**Authors:** Diana I. Serrazanetti, Francesca Patrignani, Alessandra Russo, Lucia Vannini, Lorenzo Siroli, Fausto Gardini, Rosalba Lanciotti

**Affiliations:** ^1^Centro Interdipartimentale di Ricerca Industriale Agroalimentare, Università degli Studi di BolognaCesena, Italy; ^2^Dipartimento di Scienze e Tecnologie Agro-alimentari, Università degli Studi di BolognaCesena, Italy; ^3^Servizio Sanitario Regionale, Azienda Unità Sanitaria Locale di ImolaImola, Italy

**Keywords:** *Saccharomyces bayanus*, wine yeasts, high-pressure homogenization, sub-lethal stress, membrane fatty acid changes

## Abstract

**Aims:** The aim of this work was to study the responses of *Saccharomyces bayanus* cells exposed to sub-lethal high-pressure homogenization (HPH) and determine whether the plasmatic membrane can sense HPH in the presence, or absence, of exogenous unsaturated fatty acids (UFAs) in the growth medium.

**Methods and Results:** High-pressure homogenization damaged and caused the collapse of cell walls and membranes of a portion of cells; however, HPH did not significantly affect *S. bayanus* cell viability (less than 0.3 Log CFU ml^-1^). HPH strongly affected the membrane fatty acid (FA) composition by increasing the percentage of total UFA when compared with saturated fatty acids. The gene expression showed that the transcription of *OLE1*, *ERG3*, and *ERG11* increased after HPH. The presence of exogenous UFA abolished HPH-induced effects on the *OLE1* and *ERG3* genes, increased the percentage of membrane lipids and decreased the expression of *OLE1* and *ERG3* within 30 min of treatment.

**Conclusion:** The results suggest a key role for UFA in the microbial cell response to sub-lethal stress. In addition, these data provide insight into the molecular basis of the response of *S. bayanus* to this innovative technology.

**Significance and Impact of the Study:** Elucidation of the mechanism of action for sub-lethal HPH will enable the utilization of this technology to modulate the starter performance at the industrial scale.

## Introduction

High-pressure homogenization (HPH) is one of the most encouraging alternatives to the traditional thermal treatment used for food preservation and diversification. The effectiveness of HPH in the deactivation of pathogenic and spoilage microorganisms in model and real-systems is well documented (Kheadr et al., 2002; Wuytack et al., 2002). In the food industry, high-pressure treatments are also used to modify macromolecular functional properties and obtain diversified and improved dairy products in terms of safety, texture, aroma, flavor, and shelf life. Moreover, multiple studies indicate that HPH is a useful method for cell disruption and the recovery of intracellular bio-products, including intracellular metabolites and enzymes (Shirgaonkar et al., 1998; Bury et al., 2001; Geciova et al., 2002). HPH treatment was recommended for bio-technological purposes aimed at enhancing the performance of certain lactic acid bacteria and yeasts (Tabanelli et al., 2012; Patrignani et al., 2013).

In addition, the HPH processing of certain yeast strain cell suspensions, such as *Yarrowia lipolytica* and *Saccharomyces cerevisiae*, increased the yield and inverted the enantioselectivity of the reduction of several prochiral ketones (Fantin et al., 1996). HPH has been reported as a versatile approach for the modulation of the autolytic phenomena of starter tirage cultures used for the production of sparkling wines according to the traditional method. *S. bayanus* L951 subjected to 80 MPa before the preparation of a tirage solution accelerated its re-fermentation kinetics and enhanced its autolysis phenomena and modulated the volatile molecule profile of the sparkling wine obtained with this strain (Patrignani et al., 2013). Also according to Comuzzo et al. (2015), HPH was a promising technique for inducing autolysis of wine yeasts.

Despite the versatile potential of HPH, there are limited reports focused on the response mechanisms of cells subjected to mild HPH treatments (sub-lethal stress) (Lanciotti et al., 2007; Patrignani et al., 2013). Cell membranes are the first barrier that separates microorganisms from the external environment and are a primary target for damage caused by environmental stress. Unexpected changes in environmental conditions can result in changes to the organization and dynamic structure of membrane lipids and interfere with the function of many cell activities (Rodríguez-Vargas et al., 2007; Montanari et al., 2010; Tabanelli et al., 2014). Consequently, changes in fatty acid (FA) and membrane lipid composition are fundamental in maintaining both membrane integrity and functionality after exposure to environmental stresses (Vigh et al., 2005; Di Pasqua et al., 2006; Alvarez-Ordóñez et al., 2008; Patrignani et al., 2008). Several authors have suggested a relationship between membrane composition and tolerance to several stresses, including ethanol, super-optimal temperatures, low temperature, shifts in temperature and hydrostatic pressure, freezing and salt, and sanitizing agents (Aguilera et al., 2006; Rodríguez-Vargas et al., 2007; Landolfo et al., 2010). Moreover, changes in membrane composition are reflected in the modification of physical cell surface properties (Gianotti et al., 2008), including phase transition temperature and microviscosity (Mejia et al., 1999). While studying the adaptation to H_2_O_2_, Pedroso et al. (2009) found several significant changes in the lipid profile and microdomains of the plasma membrane of *S. cerevisiae* that were associated with the expression of genes encoding key lipid biosynthesis enzymes. With regard to ethanol and acetic acid tolerance, several authors have reported a key role for zinc in the ability of *S. cerevisiae* to regulate the biosynthesis of phospholipids by modulating the gene expression of key enzymes (Carman and Han, 2007, 2011; Lei et al., 2007; Mira et al., 2010; Zhao and Bai, 2012). Moreover, an increase in the level of UFAs and changes in the FA to protein ratio is reported as adaptation mechanisms to low temperatures (Cossins et al., 2002). Multiple studies have shown that compositional changes in unsaturated fatty acids (UFAs) in various organisms were regulated by either the transcriptional or post-transcriptional modification of desaturases (Cossins et al., 2002). An increase in the UFAs percentage within yeast cells was correlated with a decrease in the responsiveness of the stress response promoter element (STRE)-driven gene to heat and salt stresses in *S. cerevisiae* (Chatterjee et al., 2001). A vital role in microbial homeoviscous adaptation has been attributed to membrane-bound desaturases, and these enzymes are crucial for synthesizing UFAs (Vigh et al., 1998; Guerzoni et al., 2001; Wallis et al., 2002).

Approximately 70–80% of the total glycerolipid acyl chains consist of the Z (*cis*)-9 species C14:1, C16:1, and C18:1 in *S. cerevisiae* cells grown under a wide range of physiological conditions. The monounsaturated FAs in *Saccharomyces* and other fungi are formed from saturated fatty acyl CoA precursors by the OLE1p Δ9-FA desaturase, an endoplasmic reticulum enzyme system that introduces the double bond into saturated fatty acyl CoA substrates. Consequently, *OLE1* gene expression is highly regulated and responds to a number of different stimuli, including carbon source, nutrient FAs, metal ions, and oxygen levels (Martin et al., 2007).

Thus, the current study sought to determine the mechanisms involved in the response of *S. bayanus* to sub-lethal HPH, a microbial species endowed with a great applicative potential oenological field as starter culture. The comprehension of the action mechanisms of sub-lethal HPH treatment is fundamental for the optimization of tirage solution performances used for the production of traditional sparkling wine at the industrial level. This also contributes to a fully understanding of the previously observed modifications of *S. bayanus* technological performances, i.e., the acceleration of autolysis phenomena and improving of sparkling wine sensorial features (Patrignani et al., 2013; Comuzzo et al., 2015), thus reducing the aging period, and consequently increasing the process sustainability as well as the product quality and differentiation. In particular, the effects of sub-lethal HPH on *S. bayanus* cells grown in the presence, or absence, of exogenous UFAs were studied. These data will allow for a better understanding of the compositional changes of UFAs in the cell membrane and the expression of desaturases, which are crucial enzymes for FA synthesis, involved in the stress response to hyperbaric treatment. The changes in membrane FA composition and the expression of genes related to stress and membrane lipid synthesis were analyzed immediately after sub-lethal HPH and during the subsequent 100 min of incubation at 28°C. The morphological changes due to the sub-lethal HPH were assessed within 24 h because this treatment is reported to modify the outer structure of microbial cells and the corresponding functional properties (Patrignani et al., 2013).

## Materials and Methods

### Strain and Experimental Procedures

All of the experiments used *S. bayanus* L951 obtained from the Department of Agricultural and Food Sciences (DISTAL, University of Bologna). The strain was isolated from Lambrusco grapes. Two experiments were designed to examine the response of *S. bayanus* to HPH treatment in the presence or absence of C18:1 (Tween 80) and C16:1 (Tween 40) in the growth medium. The strain was cultured under shaking in Sabouraud medium (Oxoid, Basingstoke, UK) at 28°C for 15 h before HPH treatment. The growth conditions were the same used by Patrignani et al. (2013).

When required, Tween 40 and Tween 80 were added to the growth medium at a concentration of 0.5 ml l^-1^.

### HPH Treatment

A PANDA high-pressure homogenizer (Niro Soavi, Parma, Italy) was used for all homogenizing treatments. The initial cell load before HPH treatment was about 7.7 log CFU ml^-1^. The control treatment was performed at 0.1 MPa, and the mild treatment was performed at 80 MPa. The machine was supplied with a homogenizing PS-type valve with a flow rate of 10 l h^-1^. The valve assembly included a ball-type impact head made of ceramic, a stainless steel, large-inner-diameter impact ring and a tungsten carbide passage head. The inlet temperature of the cell suspension was 40°C, and the rate of temperature increase was approximately 2°C 10 MPa^-1^. During the treatment, the cells remained under pressure for a few milliseconds. Immediately after the treatment at 0.1 and 80 MPa, the cell loads were verified by plating the cell suspension on Sabouraud agar medium (Oxoid, Basigstone, UK) after sonication with a Liarre Starsonic 90 to separate cell clumps.

### Growth Kinetics

*Saccharomyces bayanus* L951 was inoculated (5 log CFU ml^-1^) in Sabouraud medium (Oxoid, Basingstoke, UK) and incubated at 28°C. Cells grown to the late exponential phase in Sabouraud medium with or without exogenous UFAs were subjected to HPH treatment at 80 and 0.1 MPa (treated and untreated) and subsequently inoculated in fresh Sabouraud medium and incubated at 28°C. The growth kinetics were measured with a Burker counting chamber after sonication with a Liarre Starsonic 90 to separate cell clumps.

### Scanning Electron Microscopy

Cell morphology following HPH treatment was investigated using scanning electron microscopy (SEM) according to a previously developed method (Ndagijimana et al., 2006). *S. bayanus* L951 cells (about 7.7 log CFU ml^-1^) were subjected to 0.1 (controls) or 80 MPa, and samples were collected immediately and at 30 min, 90 min, 210 min, and 24 h after treatment.

### Lipid Extraction and Analysis of Cellular FAs

The cells were harvested 100 min after HPH treatment (0.1 and 80 MPa) by centrifugation (10 min at 8000 × *g*) and washed twice with saline solution (NaCl 0.9%). Lipid extraction and membrane FA analyses were performed according to a previously described method (Suutari et al., 1990). The lipid pool was fractioned by solid-phase extraction (SPE). For GC analyses, a GC-Mass Agilent 6890 gas chromatograph (Agilent Technologies, Palo Alto, CA, USA) coupled to an Agilent 5970 mass selective detector operating in electron impact mode (ionization voltage 70 eV) was used. The column used was a Chemtek RTX-2330 (260°C: 30 m × 250 μm × 0.2 μm; 10 cyanopropyl-90 biscyano: 236.56493). The injector and detector were both held at 250°C. The temperature was programmed from 120°C (held for 5 min) to 215°C at a rate of 4°C min^-1^, then from 215°C to 225°C at a rate of 0.5°C min^-1^, and the final temperature was held for 5 min. The carrier gas was helium with a rate of 1 ml min^-1^ and a split ratio of 1:10. Compounds were identified using the National Institute of Standards and Technology—United States Environmental Protection Agency—National Institute of Health (NIST/EPA/ NIH—Version 1998) and Wiley-Version 1996 mass spectra database. The relative percentages of the FAs were determined from the peak areas of the methyl esters using a DP 700 integrator (Spectra Physics). The unsaturation level was calculated as [% monounsaturated + 2 (% di-unsaturated) + 3 (% tri-unsaturated)/100]. The results are expressed as the mean of three independent experiments. The coefficients of variability are expressed as the percentage ratios between the standard deviations and the mean values and ranged between 2 and 5%.

### RNA Extraction, Reverse Transcription, and Gene Expression

Frozen cells were mechanically disrupted using a ball mill (Mikro-Dismembrator S; B. Braun Biotech International). Total RNA was extracted from the cells after treatment (5, 10, 20, 30, 60, and 100 min) at 0.1 and 80 MPa using an RNeasy mini kit (Qiagen). To eliminate genomic DNA contamination, an additional DNase treatment was performed according to the RNeasy kit instruction with the RNase-free DNase set (Qiagen).

The RNA concentration and purity were optically determined with a GeneQuant RNA/DNA calculator (Amersham Pharmacia Biotech, Piscataway, NJ, USA). Based on the final concentrations, 1 μg of RNA was used to obtain cDNA with 2 μg of random primers (Promega), 40 μM deoxynucleoside triphosphates (dNTPs) (Qbiogene), 4 μl reverse transcription (RT) buffer (Promega), 0.5 U Moloney murine leukemia virus (MMLV) reverse transcriptase, and an RNase H^-^ point mutant (Promega) in a total volume of 20 μl. mRNA samples were also prepared without reverse transcriptase as a control for DNA contamination. The sequences of the primers used as the housekeeping gene (actin gene – ACT1) for *S. bayanus* were as follows: ACT1_Fw, 5′-GCTGTCTTCCCATCTATCGTCG-3′; and ACT1_Rv, 5′- GGACAAAACGGCTTGGATGG-3′.

The primers were designed using *S. bayanus* genome sequences strain 623-6C. The genes selected have an important role in FA biosynthesis and are reported in **Table 1** (FAR: fatty acid regulated). In particular, YGL055W (OLE1) codes for desaturase delta-9, and YLR056W (ERG3) encodes a C-22 sterol desaturase. Both of these genes are involved in cellular FA biosynthesis. A similar regulatory motif is the gene YHR007C (ERG11) that encodes a C-14 demethylase involved in ergosterol biosynthesis. HSP70 and MPK1 were analyzed because their expression is strictly correlated with the physical properties of the membrane. Gene expression levels were determined fluorometrically with SYBR^®^ green (Lonza Rockland, Inc.) and Rotor Gene 6000 (Corbett, Australia). For each primer pair, amplification efficiency was calculated as *E* = –1 + 10^(-1/slope)^. Samples were examined for differences in gene expression using a relative quantification (relative gene expression, RGE) (Pfaﬄ, 2001; Fleige et al., 2006; Serrazanetti et al., 2011).

**Table 1:** The systematic name, standard name, and functions of the target genes and related primers used in this study.

Systematic name (standard name)	Primer	Sequence (5′→3′)	Biological function
YFL039C (*ACT1*)	ACT1-Fw ACT1-Rv	gctgtcttcccatctatcgtcg ggacaaaacggcttggatgg	Actin; structural protein involved in cell polarization, endocytosis, and other cytoskeletal functions

YGL055W (*OLE1*)	OLE1-Fw	ttccaaaggatgactctgccag	Delta-9 monounsaturated fatty acid (FA) desaturase
	OLE1-Rv	ccagttcaaatgttggtgccag	

YLR056W (*ERG3*)	ERG3-Fw ERG3-Rv	caagtggcagaaattgctaggg ccatggaacggtcaacatcg	C-5 sterol desaturase. Catalyzes the introduction of a C-5(6) double bond into episterol, a precursor in ergosterol biosynthesis

YHR007C *ERG11*)	ERG11-Fw ERG11-Rv	tgttgcacttggctgaaagacc ccttagaaatggcaccgaaacc	Lanosterol 14-alpha-demethylase. Catalyzes the C-14 demethylation of lanosterol to form 4,4″-dimethyl cholesta-8,14,24-triene-3-beta-ol in the ergosterol biosynthesis pathway

YMR015C (*ERG5*)	ERG5-Fw ERG5-Rv	aggttccatcgcaggtccaa gcaacatcgacaacgcaagg	C-22 sterol desaturase; a cytochrome P450 enzyme that catalyzes the formation of the C-22(23) double bond in the sterol side chain in ergosterol biosynthesis

YJR045C (*HSP70*)	HSP70-Fw HSP70-Rv	gctctttccgtattgccacacg cgaaacgacgaccaatcaaacg	Hsp70 family ATPase. Constituent of the import motor component of the translocase of the inner mitochondrial membrane (TIM23 complex); involved in protein translocation and folding

YHR030C (*MPK1*)	MPK1-Fw MPK1-Rv	gcatacggcatagtgtgttcagc gggcttcaaatcacgatgcaag	Serine/threonine MAP kinase; involved in regulating the maintenance of cell wall integrity, cell cycle progression, and nuclear mRNA retention in heat shock

### Statistical Analysis

Three independent replicates were performed for all experiments. To test for significant differences within the replicates, a one-way analysis of variance (ANOVA) and LSD-test were applied.

## Results

### Effects of HPH Treatment on Cell Growth and Cell Morphology

**FIGURE 1:**
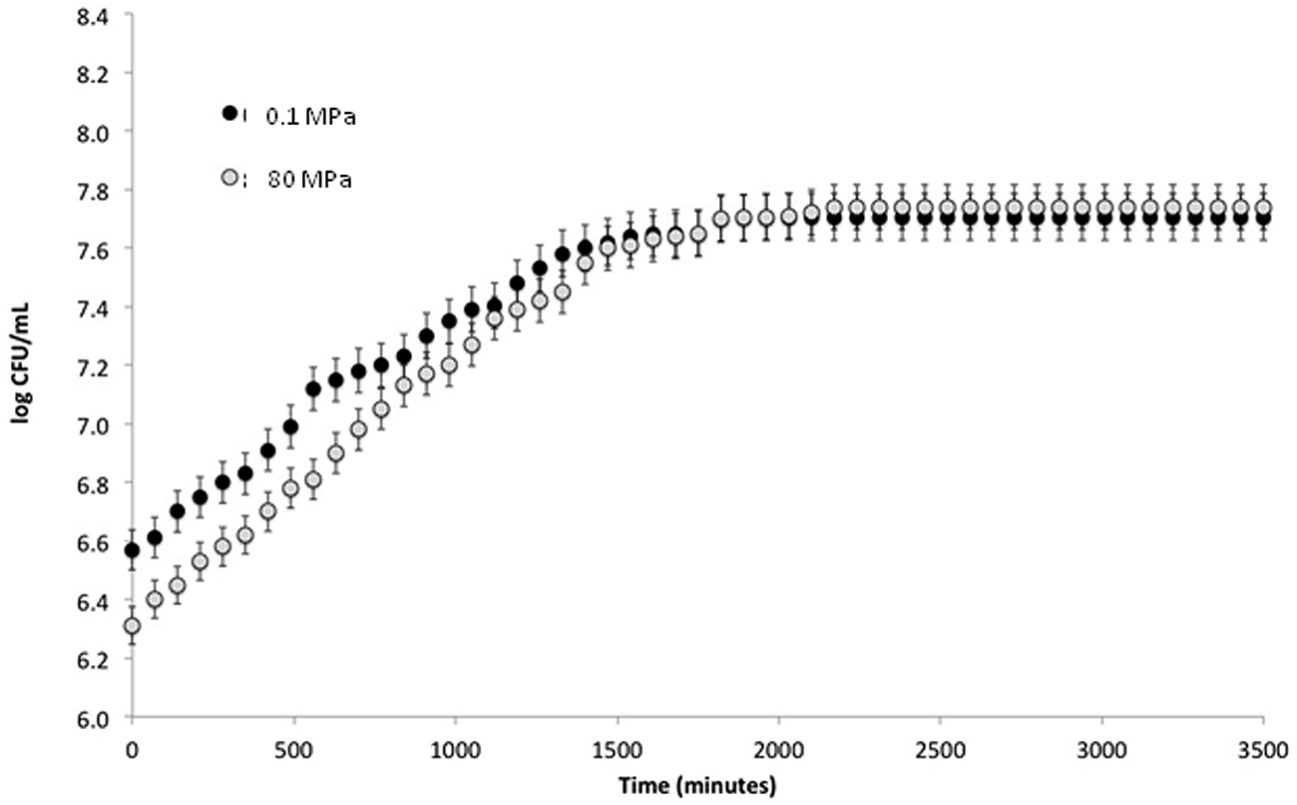
**Log CFU ml^-1^ plotted against time (min) from treatment**.

To verify the effect of HPH treatment on the viability of *S. bayanus* L951, cell load was recorded immediately after the treatment at 80 MPa and differences not exceeding 0.3 log CFU ml^-1^ were observed between the two samples (**Figure 1**).

To study the effect of HPH treatment on growth kinetics, *S. bayanus* L951 was treated at 0.1 and 80 MPa after inoculation in fresh Sabouraud medium. The cell load evolution during the incubation at 28°C was monitored using a Burker counting chamber. The growth kinetics of treated and control cells showed no significant differences after 700 min and cell loads of 7.74 ±0.67 and 7.70 ±0.45 log CFU ml^-1^, respectively, were detected after 2000 min (**Figure 1**). In addition, HPH treatment showed no modifications of the lag phase or growth rate of the cells during incubation at 28°C.

**FIGURE 2:**
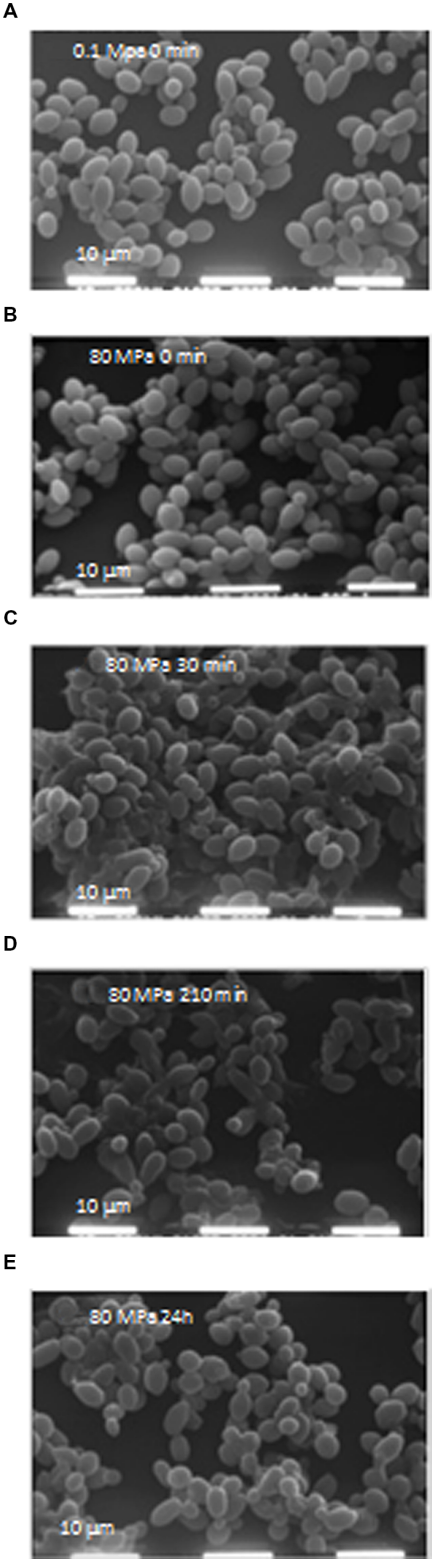
**(A–E)** Scanning electron microscopy (SEM) images. Scale: 10 μm; electron beam: 20 kV. **(A)** Cells at 0 min after 0.1 MPa high-pressure homogenization (HPH) treatment (control cells); **(B)** Cells at 0 min after 80 MPa HPH treatment; **(C)** Cells at 30 min after 80 MPa HPH treatment; **(D)** Cells at 210 min after 80 MPa HPH treatment; **(E)** Cells at 24 h after 80 MPa HPH treatment.

To verify the effects of HPH treatment on cell morphology, microscopic analyses were performed using SEM at 0, 30, 90, and 210 min as well as at 24 h. HPH treatment was performed on exponentially growing cells (0D_600_ ≈ 0.6), corresponding to a high percentage of budding cells. **Figure 2A** shows the cell morphology of the control cells (subjected to a passage through the equipment at 0.1 MPa). Based on the observation of several images of each sample (data not shown), approximately 30% of total cell population showed damages due to the application of the HPH treatment at 80 MPa. As evidenced in **Figure 2B**, damages were collapse or loss of turgor of the cell wall and membrane. The majority of cells showed an unmodified morphology and continued their growth cycle as shown as an example in **Figure 2B**. At 30 min (**Figure 2C**) and 90 min (data not shown) after the treatment, the collapsed cells decreased, while the percentage of budding cells doubled in comparison with the samples analyzed immediately after the 80 MPa treatment and the control cells (**Figures 2A,B**, respectively). At 210 min after HPH treatment, there were fewer morphologically destroyed cells, whereas several budding cells and bud scars were evident (**Figure 2D**). At this stage, HPH-treated cells were in the active multiplication phase, supporting the growth kinetics data (**Figure 1**). Twenty-four hours after HPH treatment (**Figure 2E**), the cells appeared disaggregated and in active lysis when compared with control cells after 24 h of incubation (data not shown). These morphological changes suggest that autolysis was stimulated by HPH treatment.

### Effects of HPH Treatment on FA Composition in the Cell Membrane

**Table 2** shows the FA composition of the *S. bayanus* control and HPH-treated cells. The analyses were performed at 100 min after the HPH treatment. The main FAs detected in both control and HPH-treated cells were C16:0, C18:1Δ9*cis* and C16:1(Δ9)*cis*. The HPH treatment strongly increased the percentage of total UFAs with a proportional reduction of the saturated fattty acid (SFA) percentage; moreover, the UFA/SFA ratio significantly increased from 0.7 ± 0.2 to 1.6 ± 0.3 (**Table 2**). In the 80 MPa treated cells, the 16:1/16:0 and 18:1/18:0 ratios increased, respectively, from 0.4 ± 0.1 to 1.5 ± 0.4 (16:1/16:0) and from 3.2 ± 0.7 to 6.7 ± 1.4 (18:1/18:0) with respect to cells treated at 0.1 MPa. The unsaturation level increased from 41.1% at 0.1 MPa to 61.2% at 80 MPa. In particular, the unsaturation level increased due to the significant enhancement of C16:1 and C18:1 (**Table 2**).

**Table 2:** Relative percentages of FA ethyl esters in cell membranes in relation to high-pressure homogenization (HPH) treatment (0.1 or 80 MPa).

															FA ethyl esters (percentage)					
**Condition (MPa)**	**C8:0**	**C10:0**	**C12:0**	**C14:0**	**C14:1**	**a-C15**	**C16:0**	**C16:1 (7)**	**C16:1 (9)**	**C17:0**	**C18:0**	**C18:1 (9) *cis***	**C18:1 (10) *cis*/*trans***	**C18:2**	**UFA**	**SFA**	**UFA/SFA**	**16:1/16:0**	**18:1/18:0**
0.1	–	0.48 ± 0.24	2.88 ± 0.25	7.00 ± 0.12	2.42 ± 0.32	2.57 ± 0.32	38.23 ± 8.35	5.20 ± 1.60	9.71 ± 1.26	1.25 ± 0.22	5.86 ± 0.01	17.72 ± 3.71	1.53 ± 0.48	4.60 ± 0.46	41.1 ± 7.5	57.2 ± 10.0	0.7 ± 0.2	0.4 ± 0.1	3.2 ± 0.7

80	0.07	1.20 ± 0.09	3.56 ± 0.24	7.11 ± 0.09	1.54 ± 0.48	–^∗^	22.42 ± 2.27	1.30 ± 0.22	33.67 ± 5.79	0.08 ± 0.02	3.60 ± 0.33	23.55 ± 3.03	0.58 ± 0.06	0.62 ± 0.06	61.2 ± 9.6	38.4 ± 3.0	1.6 ± 0.3	1.5 ± 0.4	6.7 ± 1.4

*The data are shown as the mean and relative SD from three independent experiments and two GC–MS determinations. The coefficients of variability were lower than 5%.*

*^∗^Under the detection limit.*

### Effects of HPH Treatment on the Expression of Genes Involved in Lipid Metabolism

The genes reported in **Table 1** were selected based on their involvement in lipid synthesis. The purpose of the gene expression study was to verify whether those genes are the effectors of the changes in the UFA/SFA ratio and of cell responses related to membrane FA modification after sub-lethal HPH treatment.

**FIGURE 3:**
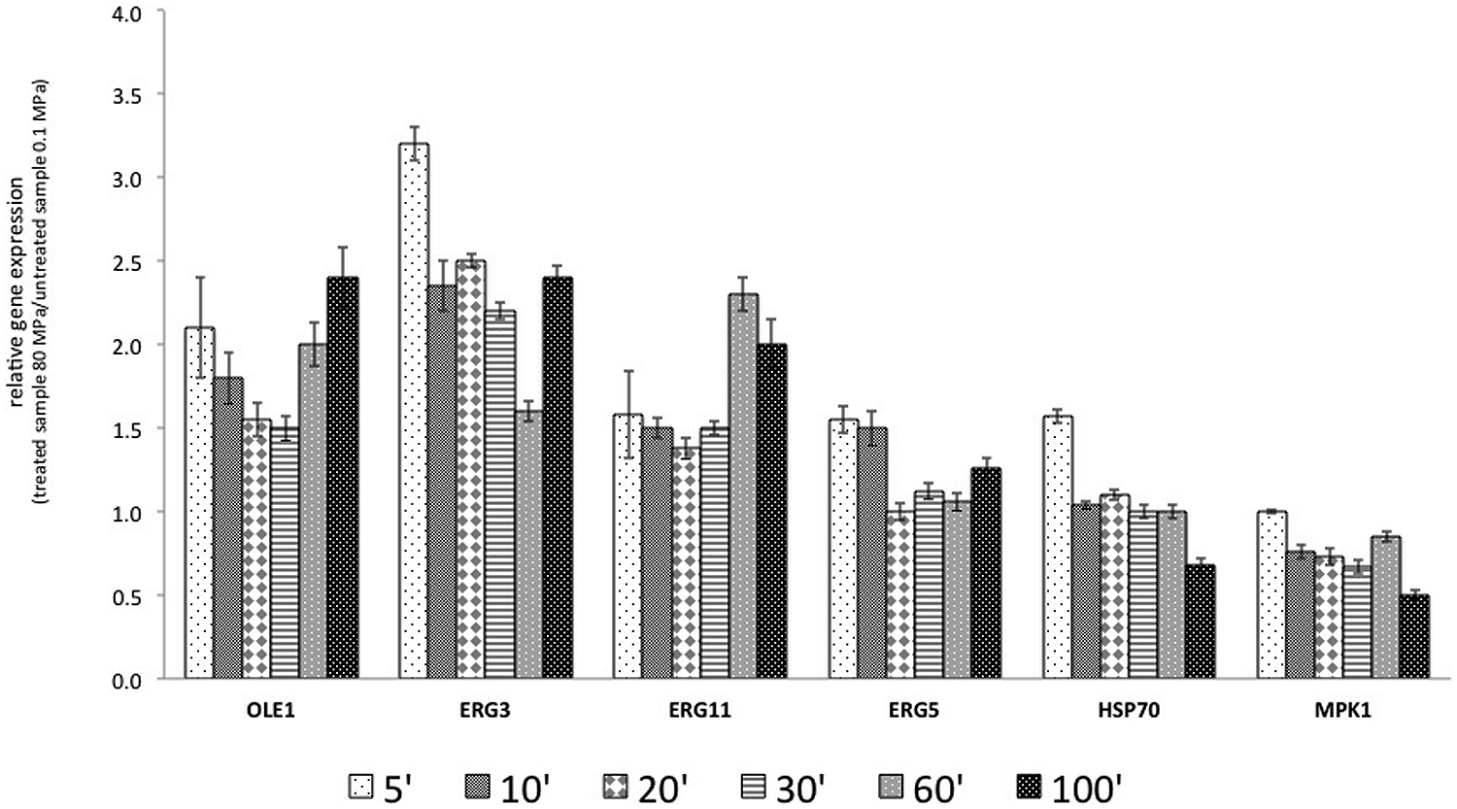
**Relative gene expression (RGE) calculated with the samples treated at 0.1 MPa, as a control, and samples treated, at 80 MPa representing sub-lethal stress at 5, 10, 20, 30, 60, and 100 min after the start of treatment.** The gene products included *OLE1*, a delta-9 monounsaturated fatty acid (FA) desaturase; *ERG3*, a C-5 sterol desaturase; *ERG11*, a lanosterol 14-alpha-demethylase; *ERG5*, a C-22 sterol desaturase; *HSP70*, an hsp70 family ATPase; and *MPK1*, a serine/threonine MAP kinase. The reported values are statistically significant (*p* < 0.01).

The results reported in **Figure 3** show that the transcription of OLE1 increased after HPH treatment. In particular, after 5 min of the treatment, the transcript increased by 100%. The overexpression of *OLE1* was constant for all analyzed times: 100% after 5 min, 50% after 20–30 min, and again 100% after 60 and 100 min. *ERG11* showed a similar behavior. The transcription of *ERG3*, which encodes ergosterol desaturase (C-5 sterol desaturase), was markedly and rapidly activated after treatment (threefold). The gene remained overexpressed throughout the course of the experiment. *ERG5* encodes for the sterol desaturase C-22 and was overexpressed immediately after treatment (5 and 10 min from the start of treatment).

According to the experimental results, the target genes were overexpressed after HPH treatment. Moreover, *OLE1*, *ERG3*, and *ERG11* presented a similar behavior, with an inflection of the overexpression in the central times analyzed (20–30 min for *OLE1* and *ERG11* and 60 min for *ERG3*). A different behavior was observed for *HSP70*. This gene was overexpressed beginning at 5 min from the start of treatment and then subsequently decreased. HPH treatment reduced the expression of *MPK1*.

### Effects of HPH Treatment on Cell Membrane FA and Target Gene Expression in the Presence of Exogenous UFAs

The membrane FA composition and the RGE were evaluated to better understand the role of exogenous FAs, such as C16:1 and C18:1, in the response of cells to sub-lethal HPH treatment (80 MPa).

The data of membrane FAs confirmed that HPH increased the UFA concentration independently from exogenous FA supplementation. In fact, after treatment at 80 MPa, the increase in the ratio UFA/SFA was about 2.5-fold (**Table 3**).

**Table 3:** Percentages and ratios of total unsaturated fatty acid (UFA) and saturated fatty acid (SFA) ethyl esters in relation to HPH treatments (0.1 or 80 MPa) in the presence of exogenous UFAs.

Condition (MPa)	UFA	SFA	UFA/SFA	16:1/16:0	18:1/18:0
0.1	36.8 ± 4.6	60.4 ± 3.4	0.6 ± 0.1	0.3 ± 0.1	2.7 ± 0.5
80	61.9 ± 4.9	39.3 ± 4.8	1.5 ± 0.2	0.5 ± 0.1	106 ± 9.2

*The ratio of 16:1/16:0 and 18:1/18:0 after HPH is shown (0.1 or 80 MPa). The data are reported as the mean and relative SD from three independent experiments and two GC–MS determinations. The coefficients of variability were lower than 5%.*

**FIGURE 4:**
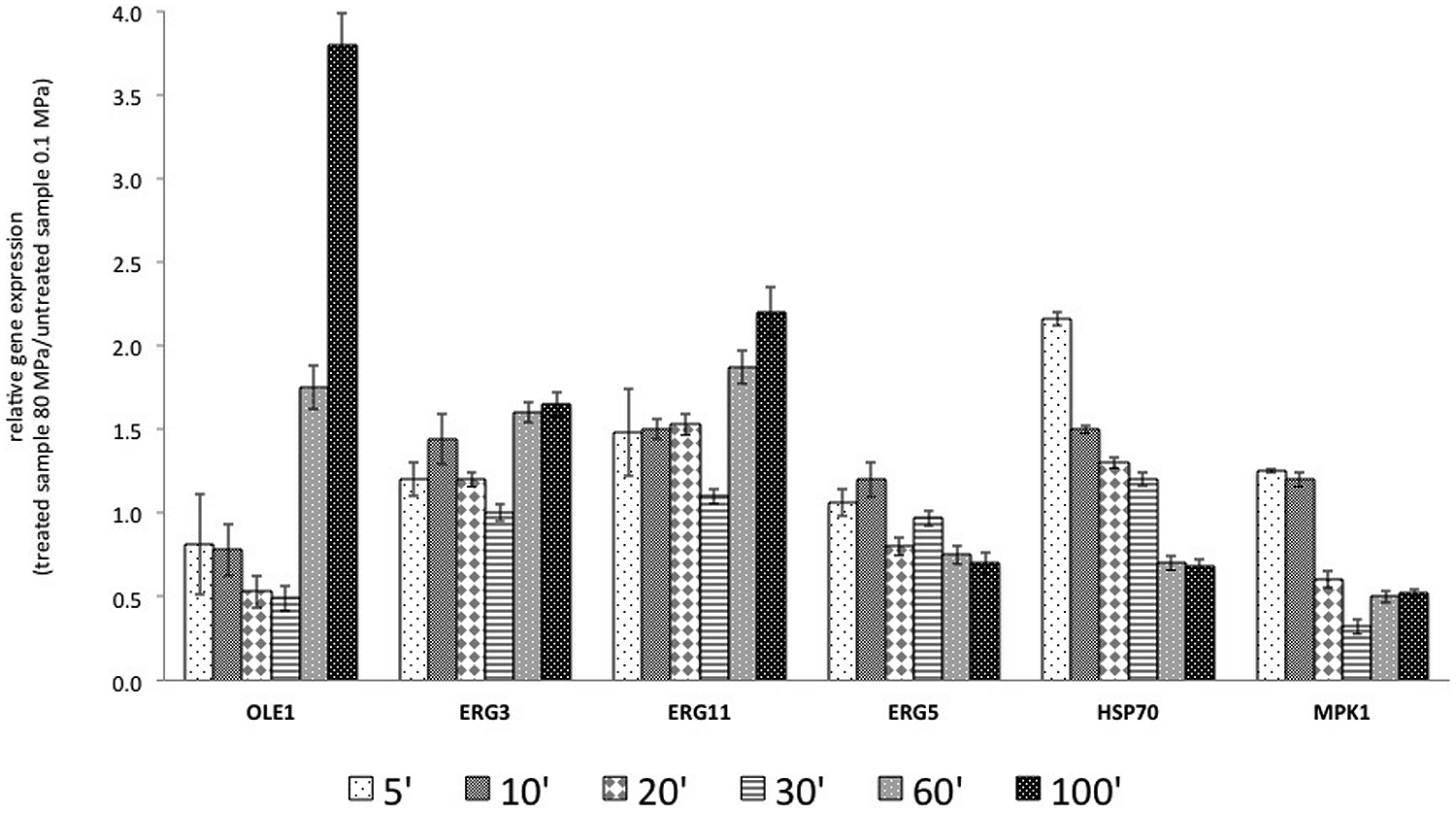
**Relative gene expression calculated with the samples treated at 0.1 MPa, as a control, and samples treated at 80 MPa, representing sub-lethal stress at 5, 10, 20, 30, 60, and 100 min after the start of treatment, in the presence of exogenous FAs (C18:1 and C16:1).** The gene products included *OLE1*, a delta-9 monounsaturated FAs desaturase; *ERG3*, a C-5 sterol desaturase; *ERG11*, a lanosterol 14-alpha-demethylase; *ERG5*, a C-22 sterol desaturase; *HSP70*, an hsp70 family ATPase; and *MPK1*, a serine/threonine MAP kinase. The reported values are statistically significant (*p* < 0.01).

The data regarding the RGE of the selected genes in the presence of exogenous UFA and after HPH treatment (80 MPa) are reported in **Figure 4**.

In the first 30 min after treatment, the transcription of *OLE1* was repressed, but after 60 and 100 min, it was increased. The presence of exogenous C16:1 and C18:1 in the growth medium of the control culture and the treated culture abolished the effects of HPH on *ERG3* observed in **Figure 3**. In fact, in the first 30 min, *ERG3* transcription was at the same levels in the cells treated at both 0.1 and 80 MPa. In all the samples grown in the presence of exogenous UFAs, a reduction in the expression of *ERG3* in the first 30 min was observed with respect to the cells not supplemented with those FAs. At 60 and 100 min, the gene *ERG3* was overexpressed, as reported in **Figure 4**. Exogenous C16:1 and C18:1 showed similar effects on *OLE1*. The transcription of *ERG11* was influenced only by the HPH treatment and not by the presence of exogenous UFAs (C16:1 and C18:1). In fact, the response of this gene to the treatment was almost the same as that reported in **Figure 3**. The RGE of the gene *ERG5* was mildly reduced in all the analyzed times. This gene showed a different transcriptional response when compared with two genes that code for desaturases (*OLE1p* and *ERG3p*). The transcription of *HSP70* was inversely correlated with the transcription of *OLE1* after HPH treatment. The transcription of *MPK1* was unmodified after HPH treatment in combination with UFA.

## Discussion

The cellular FA composition is the result of a combination of complex phenomena maintaining the optimal viability of the cell under different conditions, which makes it difficult to understand the adjustment mechanisms linking FA composition to various stress factors (Russell et al., 1995; Guerzoni et al., 2001; Gianotti et al., 2008; Serrazanetti et al., 2009; Montanari et al., 2010; Tabanelli et al., 2014). The crucial role of UFAs in microbial stress response was previously reported in yeasts and bacteria by several authors (Diefenbach et al., 1992; Brown et al., 1997; Di Russo et al., 1999; Guerzoni et al., 2001; Gianotti et al., 2008).

Sub-lethal HPH treatment causes a strong alteration of membrane FA composition in *S. bayanus* cells. In particular, the treated cells responded with morphological alterations and by increasing the proportion of UFAs in membrane FAs. This phenomenon demonstrates a clear link between the sub-lethal stress and the membrane FA response. In fact, the increase in the UFA/SFA ratio doubled in cells not supplemented with Tween 40 and Tween 80 (C16:1 and C18:1, respectively) (**Table 2**). Also the increase of temperature, up to super-optimal temperature (36–38°C) due to the HPH treatment could contribute to the unsaturation level increase. In fact, in yeasts, the crucial role of the increased unsaturation level was demonstrated in thermotolerant strains of *S. cerevisiae* when exposed to both super-optimal temperatures and oxidative stress (Guerzoni et al., 1997a). However, during the HPH treatment the exposure to super-optimal temperature is of few milliseconds and consequently is hypothesized a major role of HPH treatment in the observed FAs changes. In the cells supplemented with UFAs, the ratio UFA/SFA increased mainly due to the incorporation of the exogenous C18:1. This increase was principally determined by the dramatic increase of C18:1, accompanied by the decrease of its precursor C18:0. An increase in the proportion of oleic (*cis*-9-octadecenoic acid) acid to linoleic (*cis*-9,*cis*-12-octadecadienoic acid) acid with temperature has also been observed in thermotolerant *Hansenula polymorpha* (Wijeyaratne et al., 1986). An oxygen-dependent desaturase induction in thermotolerant yeast strains was postulated to prevent an increased accumulation of oxygen and reactive oxygen species (ROS) in the membrane at superoptimal temperatures and protect the cells from damage generated by oxidative and thermal stresses (Guerzoni et al., 1997a,b). An increase in UFAs has been reported to play a key role in maintaining the membrane in a functional liquid crystalline state (homeoviscous adaptation). Cells can control their fluidity by modulating their membrane composition to maintain an optimal level of fluidity within the lipid matrix. In fact, many deep-sea organisms modulate their membrane fluidity by increasing the proportions of UFAs in response to pressure (Bartlett, 2002). In addition, a higher proportion of UFAs in membrane lipids is correlated with resistance under high hydrostatic pressure (Bravim et al., 2010; de Freitas et al., 2012). An increase in the presence of UFAs in yeast cells was correlated with a decrease in the responsiveness of the STRE-driven gene to heat and salt stresses (Chatterjee et al., 1997, 2000).

In addition, a sub-lethal HPH treatment can generate oxidative and thermal stresses. In fact, Dodd et al. (1997) postulated that stress conditions result in an oxidative stress for the cell due to an imbalance that occurs when the survival mechanisms are unable to deal adequately with the ROS in the cells. Although HPH is regarded as a non-thermal technology, during treatment, the temperature increases due to frictional heating in the homogenization valve (Diels and Michiels, 2006; Donsì et al., 2013). The temperature rise depends on several factors, such as inlet temperature, pressure level, number of passes, matrix, valve geometry, temperature exchanger.

The results regarding the increased unsaturation level in the cells subjected to a sub-lethal HPH treatment were confirmed by the gene expression study that considered genes regulating desaturases and heat shock proteins. In particular, the genes selected were associated with FA biosynthesis (*OLE1*), ergosterol biosynthesis (*ERG3*, *ERG11*, and *ERG5*), and general stress response (*MPK1* and *HSP70*). In general, after the sub-lethal HPH treatment, the transcription level of the desaturases increased (**Figure 3**). This behavior was particularly emphasized by the overexpression of *OLE1* (delta-9 monounsaturated FA desaturase), *ERG3* (C-5 sterol desaturase), and *ERG11* (lanosterol 14-alpha-demethylase). The data regarding the increased expression of *OLE1* and the decreased expression of *HSP70* after the application of sub-lethal stress were in agreement with those reported by Carratù et al. (1996), who demonstrated an increase in UFA levels in the cell membrane as a consequence of the up-regulation of *OLE1* and a reduction of the perception of stress by the cells one hour after treatment, as a consequence of the down-regulation of *HSP70*. However, *OLE1* overexpression has been reported to enhance ethanol fermentation and ethanol tolerance in yeasts (Yamada et al., 2005). In addition, de Freitas et al. (2012) reported that the continued expression of heterologous desaturase induces an adaptive response that significantly improves cell resistance to subsequent oxidative stress.

In the present study, the responses to sub-lethal HPH treatment were also evaluated in the presence of exogenous C16:1 and C18:1. It is well known that *S. cerevisiae*, as well as other microorganisms, can import long-chain UFAs from the environment, avoiding the autogenous biosynthesis. If the cells are incubated in the presence of C16:1 and C18:1, these FAs are incorporated in the membrane lipids, and the levels of *OLE1* expression are consequently reduced (Martin et al., 2007). Moreover, the presence of UFAs in the growth medium implies a high instability of *OLE1* mRNA (Kandasamy et al., 2004). In this context, the dramatic repression of *OLE1* and *ERG3* transcription in the first 30 min after HPH treatment can be attributed to C18:1 supplementation and the increased ability of the membrane to import exogenous FAs. The higher level of these substances increased the metabolism of the cells and led to a faster consumption of exogenous FAs compared with untreated cells, with the consequent up-regulation of *OLE1* and *ERG3* after 60 and 100 min after the HPH treatment. Additionally, the same behavior shown by *OLE1* and *ERG3* may be due to the presence of the same regulatory motif (GC FAR) in the promoter sequence of these genes (*S. cerevisiae* Promoter Database at http://rulai.cshl.edu/SCPD: regulatory elements and transcriptional factors).

## Conclusion

The results of this study provide new information for understanding the role of UFAs in microbial cell resistance to sub-lethal stresses, including the regulation of genes involved in FA membrane composition and modulation, also in relation to the supplementation of exogenous C16:1 and C18:1. Moreover, a deeper knowledge of the mechanism of action of sub-lethal HPH will enable the use of this technology to modulate starter performance at the industrial scale. In fact, this technology has already been employed to improve the performance of *S. bayanus* L951 in the production of traditional sparkling wine.

## Conflict of Interest Statement

The authors declare that the research was conducted in the absence of any commercial or financial relationships that could be construed as a potential conflict of interest.
